# Effect of Porosity on the Impedance Response of Porous GaAs-Based NO_2_ Gas Sensors

**DOI:** 10.3390/s26144433

**Published:** 2026-07-13

**Authors:** Janis Peksa, Maksym Kogdas, Vladyslav Bahno, Andrii Perekrest, Dmytro Mamchur

**Affiliations:** 1 Information Technology Faculty, Turiba University, Graudu Street 68, LV-1058 Riga, Latvia; pksg13@gmail.com (A.P.); dgmamchur@gmail.com (D.M.); 2 Institute of Information Technology, Riga Technical University, Kalku Street 1, LV-1658 Riga, Latvia; 3 Computer Engineering and Electronics Department, Kremenchuk Mykhailo Ostrohradskyi National University, Universitetska Street 20, 39600 Kremenchuk, Ukraine; kogdasmax@gmail.com (M.K.); vladislavbagnu@gmail.com (V.B.)

**Keywords:** porous GaAs, NO_2_ gas sensor, impedance spectroscopy, porosity optimization, constant phase element, hysteresis, nonlinear response, semiconductor gas sensors, frequency-dependent sensing

## Abstract

**Highlights:**

**What are the main findings?**

- Porosity strongly influences the impedance response of porous GaAs sensors, with maximum sensitivity observed at an intermediate porosity of ~52–53%.
- The NO_2_ sensing response is frequency-dependent, with the highest sensitivity occurring in the low-frequency range (5–10 kHz).

**What are the implications of the main findings?**

- Impedance spectroscopy provides a physically informative approach for analyzing morphology–response relationships in semiconductor gas sensors.
- Porous GaAs is a promising platform for impedance-based sensing, enabling investigation of nonlinear and hysteretic gas–surface interactions.

**Abstract:**

Nitrogen dioxide (NO_2_) is a hazardous atmospheric pollutant that requires reliable detection technologies with high sensitivity and clear physical interpretability. In this study, the influence of porosity on the impedance response of porous gallium arsenide (GaAs)-based gas sensors was systematically investigated under NO_2_ exposure. A series of samples with controlled porosity in the range of 10–70% was fabricated by electrochemical etching and analyzed using impedance spectroscopy in the frequency range of 1–100 kHz. The results show that increasing porosity enhances impedance dispersion and gas sensitivity, particularly in the low-frequency region of 5–10 kHz, where the response to NO_2_ is most pronounced. The concentration dependence of the impedance magnitude exhibits nonlinear sigmoidal behavior, while the maximum relative sensitivity is observed at an intermediate porosity of approximately 52–53%. This indicates a balance between adsorption capacity and electrical conductivity. These findings show that porosity is a key parameter governing the impedance-based sensing behavior of the porous GaAs and support the use of frequency-domain analysis for studying morphology–response relationships in semiconductor gas sensors.

## 1. Introduction

Nitrogen dioxide (NO_2_) is one of the most hazardous gaseous pollutants in urban, industrial, and transport-related environments. Due to its strong oxidizing character and pronounced toxicity, even at relatively low concentrations, continuous NO_2_ monitoring remains an important task in environmental control, industrial safety, and smart sensing systems [1,2,3,4,5]. This requirement has driven the development of gas sensors capable of operating with low detection limits, fast response and recovery, stable operation, and acceptable selectivity under practical conditions.

In recent years, the dominant progress in NO_2_ sensing has been achieved using metal oxides, graphene-derived materials, transition-metal chalcogenides, MXene-based composites, and other hybrid nanostructures [1,2,3,4,5], which demonstrated substantial improvements in sensitivity and, in some cases, room-temperature operation down to the ppb range. However, this performance is often achieved through complex heterostructure engineering, external activation methods, or highly optimized operating conditions, which may complicate interpretation of the underlying sensing mechanism and limit direct comparison between studies [1,3,4,5]. Furthermore, most high-performing NO_2_ sensors are still evaluated through DC resistance changes. This single-point metric is limiting. It cannot reveal the multiple coupled processes underlying the sensor’s response, such as adsorption-induced charge transfer, interfacial barrier modulation, or morphology-dependent transport effects.

Impedance spectroscopy provides a broader analytical framework for the study of such systems. Unlike single-point resistance measurements, impedance-based characterization enables the separation of the contributions related to charge-transfer resistance, interfacial polarization, non-ideal capacitance, and frequency-dispersive transport phenomena [6,7,8,9]. This capability is especially important for heterogeneous porous sensing materials, in which the electrical response is not governed by a single conduction mechanism. Therefore, for gas sensors that are based on porous semiconductors, impedance spectroscopy is not only a transduction method, but also a tool for mechanistic interpretation, as it can clearly reveal whether the gas exposure predominantly affects interfacial barriers, distributed capacitance, bulk transport, or several processes simultaneously [6,7,8,9].

Porous semiconductors have long attracted attention as gas-sensing materials because porosity enhances the accessible adsorption surface and modifies carrier transport near the surface or across semiconductor interfaces [10,11]. Within this broad class of materials, porous GaAs represents a particularly interesting but insufficiently explored platform. GaAs is a III–V compound semiconductor with a direct bandgap of approximately 1.42 eV at room temperature, high electron mobility, and surface/interface electronic states that can be affected by adsorbed species. In porous form, these properties may be combined with increased surface area and morphology-controlled charge transport. At the same time, the presence of arsenic requires controlled fabrication, handling, and waste-management procedures, which partly explains why porous GaAs has been less widely explored than conventional metal-oxide sensing materials. Previous studies demonstrated that porous-GaAs-based junctions can respond to gaseous species and that the morphology of the porous layer can strongly affect their electrical characteristics [12,13,14,15]. In particular, Au/porous-GaAs Schottky structures were shown to exhibit high sensitivity to polar gases such as CO and NO [12], while subsequent work confirmed that gas adsorption influences the impedance of porous GaAs [14] and that porous-layer morphology affects the parameters of Pd/porous-GaAs Schottky contacts [15].

Despite these promising early indications, the literature on porous GaAs gas sensors is sparse compared to the extensive research on metal oxides or 2D hybrids [1,2,3,4,5,10]. The existing studies largely focus on DC electrical characteristics or general gas adsorption [12,14,15]. Consequently, the relationship between the material’s porosity, its complex impedance response and the emergence of nonlinear or hysteretic effects remains poorly understood. These issues are particularly relevant because porosity is expected to influence not only the number of active adsorption sites, but also the distribution of interfacial environments, the effective transport pathways, and the non-ideal capacitive behavior of the sensing structure.

Interpreting impedance data from porous sensing materials requires robust equivalent-circuit modeling. Ideal RC models are usually insufficient for porous and heterogeneous systems because they cannot represent distributed relaxation and non-ideal capacitive behavior. Such behavior is commonly described using constant phase elements (CPEs) [7,8,9,16,17]. The use of CPEs is well established in impedance analysis, especially for rough, chemically heterogeneous, or distributed interfaces [7,16,17]. However, the physical interpretation of fitted circuit parameters in gas sensors remains challenging, as multiple models may describe the same spectrum with comparable statistical quality [6,7,8,9]. For this reason, equivalent-circuit analysis will be most convincing when supported by structural arguments, gas-response trends, and systematic variations in fitted parameters under controlled changes in morphology or gas exposure. In the case of porous GaAs, such a morphology-informed impedance interpretation is still missing in the literature.

Nonlinear response and hysteresis are also important in NO_2_ sensing. They may arise from differences between adsorption and desorption pathways, gradual filling of surface or interface states, competitive adsorption, and slow relaxation of barrier-controlled transport [4,18,19]. In porous materials, these effects can be intensified because pores introduce a distribution of adsorption environments and transport lengths. Recent NO_2_ studies on CNT-based devices and impedimetric porous platforms show that response and recovery may involve multiple time constants rather than a single reversible process [18,19,20]. For porous GaAs, such nonlinear and hysteretic effects have not yet been sufficiently connected to porosity-dependent impedance behavior.

This study investigates how the morphology of porous GaAs, specifically its degree of porosity, influences the impedance characteristics of gas-sensing structures under NO_2_ exposure. The work focuses on frequency-dependent impedance response, concentration dependence, equivalent-circuit interpretation, and nonlinear or hysteretic behavior.

The main contributions are:

(i) Identification of an intermediate porosity range of approximately 52–53% at which the relative NO_2_ response is maximized;
(ii) Demonstration that the most informative sensing response occurs in the low-frequency region of approximately 5–10 kHz within the measured range;
(iii) Analysis of nonlinear and hysteretic behavior as morphology-dependent response features;
(iv) Impedance-based interpretation using an equivalent-circuit model with a CPE-based description of non-ideal interfacial behavior.

By linking porous morphology with impedance-derived parameters, the study addresses a specific gap in porous GaAs gas sensing and provides a basis for more physically interpretable semiconductor NO_2_ sensors.

## 2. Materials and Methods

### 2.1. Automated Measurement System Architecture

**Figure 1:**
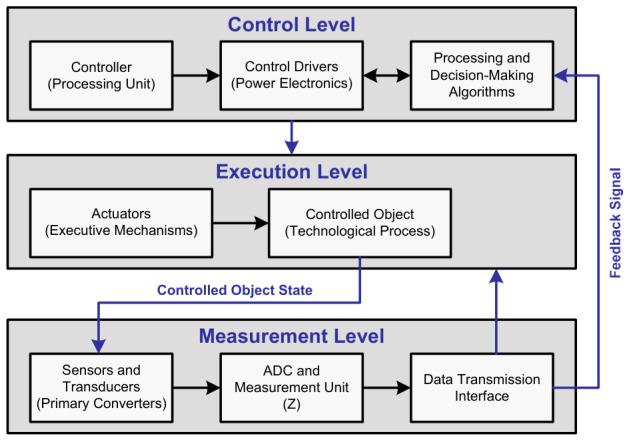
Schematic diagram of the automated measurement system used for impedance-based NO_2_ sensing experiments. The system integrates gas delivery, environmental control, the gas chamber with porous GaAs sensing element, impedance measurement, and data acquisition modules.

A dedicated automated measurement system was developed to enable synchronized control of the gas environment and multi-frequency impedance acquisition. The system follows a modular architecture integrating gas delivery, environmental control, impedance measurement, and data acquisition (Figure 1).

The sensing element consisted of a planar GaAs sample (10 × 10 × 0.5 mm) with AuGe/Ni/Au ohmic contacts, mounted in a hermetic ceramic holder with platinum spring contacts to ensure stable electrical coupling. The sensor was placed inside a stainless-steel gas chamber (50 cm^3^) equipped with gas inlet and outlet ports, a type-K thermocouple (±0.5 °C), and an absolute pressure sensor (±0.1% FSO).

Gas mixtures were generated using two mass flow controllers (EL-FLOW Select, Bronkhorst High-Tech B.V., Ruurlo, The Netherlands) for nitrogen (N_2_) and NO_2_ (1000 ppm in N_2_), enabling precise concentration control within the range of 0–100 ppm at a total flow rate of 200 ± 2 mL·min^−1^. Temperature stabilization was achieved using a resistive heater controlled by a PID loop implemented in a programmable logic controller (Siemens S7-1200, Siemens AG, Munich, Germany).

Impedance measurements were performed using a modified AD5933-based analyzer (AD5933, Analog Devices, Inc., Norwood, MA, USA), operating in the frequency range of 1–100 kHz with an excitation amplitude of 500 mV. The standard calibration path was replaced with a direct connection to the sensor cell. For each frequency point, 64-fold averaging of the discrete Fourier transform (DFT) output was applied to improve measurement stability. The measurement accuracy was validated through calibration and repeated measurements, ensuring reliability within the investigated impedance range.

System-level coordination, data acquisition, and preprocessing were implemented using LabVIEW 2026 (National Instruments Corp., Austin, TX, USA), which enabled automated execution of measurement cycles, real-time monitoring, and structured data logging. The integration of gas control and impedance measurement within a single automated framework ensured reproducible experimental conditions and enabled systematic investigation of frequency-dependent sensor response.

### 2.2. Materials and Sample Preparation

Planar GaAs specimens with nominal dimensions of 10 × 10 × 0.5 mm were used as the sensing substrates.

The starting substrates were commercially available chromium-doped semi-insulating GaAs monocrystalline wafers with a nominal (100) crystallographic orientation. According to the supplier specifications, the wafers had a diameter of 92 mm, a thickness of 400 μm, a chromium impurity concentration of approximately 5 × 10^14^ cm^−3^, and a specific resistivity of about 10^7^ Ω·cm. Planar samples with dimensions of 10 × 10 mm were cut from the wafers and used for subsequent electrochemical processing.

Porous GaAs layers were fabricated by anodic electrochemical etching. The etching electrolyte was prepared immediately before use by mixing aqueous hydrofluoric acid solution (HF, 48 wt.%) with absolute ethanol (C_2_H_5_OH, purity ≥ 99.8%) in a volume ratio of 1:3. The anodization current density was varied from 10 to 75 mA·cm^−2^, while the electrolyte temperature was maintained at 22 ± 1 °C. All procedures involving HF were carried out under appropriate laboratory safety conditions.

For quantitative characterization of the porous morphology, low-temperature nitrogen adsorption–desorption measurements were performed using a Micromeritics ASAP 2020 analyzer (Micromeritics Instrument Corp., Norcross, GA, USA). The specific surface area was calculated using the Brunauer–Emmett–Teller (BET) method. The pore-size distribution and average pore diameter were estimated using the Barrett–Joyner–Halenda (BJH) method from the desorption branch of the isotherm. The total pore volume was determined from the amount of nitrogen adsorbed at a relative pressure of (*p*/*p*_0_ = 0.99). The adsorption-derived parameters were in good agreement with SEM observations, confirming the reliability of the structural characterization of the porous GaAs samples.

An unetched monocrystalline sample (MC) served as the reference, while seven porous samples were prepared to span a broad morphology range. In the present study, porosity, denoted as P, was treated as the primary controlled structural parameter and defined as the ratio of pore volume to the total porous-layer volume. The investigated sample set covered nominal porosity levels from approximately 10% to 70%, thereby enabling analysis of both low-porosity and highly developed porous morphologies. The corresponding structural descriptors for the sample series included specific surface area, average effective pore diameter, porous-layer thickness, effective dielectric constant, and contact resistance (Table 1). The corresponding structural descriptors for the sample series included specific surface area, average effective pore diameter, porous-layer thickness, effective dielectric constant, and contact resistance.

The reference monocrystal exhibited negligible porosity and a low specific surface area, whereas the porous samples showed a monotonic increase in specific surface area from 8.7 ± 0.5 to 142.5 ± 4.5 m^2^/g as porosity increased from 10.2 ± 1.5% to 68.9 ± 3.0%. Over the same range, the average effective pore diameter increased from 18 ± 3 to 35 ± 7 nm, while the effective dielectric constant decreased from 11.35 ± 0.15 to 5.47 ± 0.50. These changes are important because they indicate that increasing porosity altered not only the available adsorption area, but also the electrical heterogeneity of the material. The porous-layer thickness remained of the same order across the porous series, varying from approximately 430 to 480 nm according to the values represented in Table 1.

**Figure 2:**
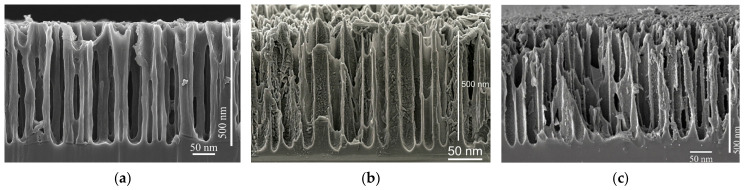
SEM cross-section images of representative porous GaAs structures with different porosity levels: (**a**) P10, approximately 10% porosity; (**b**) P50, approximately 52% porosity; (**c**) P70, approximately 69% porosity. The images illustrate the evolution of the porous layer morphology with increasing adonization current density. Scale bars are shown in each panel.

The morphology of the obtained porous structures was confirmed by scanning electron microscopy (SEM) on the cross-sections of the samples. Figure 2 shows SEM images for three representative samples with different degrees of porosity: P10 (10%), P50 (52%), and P70 (69%). The measured values of the porous layer thickness (*d*_layer_) and the average pore diameter (*d*_por_) are in good agreement with the data in Table 1. With an increase in the anodic current density, an increase in the pore diameter and a slight decrease in the layer thickness are observed, which is associated with the intensification of lateral etching at high current densities and partial erosion of the surface layer [21,22]. The obtained SEM images additionally verify the morphological evolution: from uniform porosity (P10) to the optimal percolation structure (P50) and boundary stability (P70).

Reference frequency dependencies of impedance $Z_{0}(f)$ for all eight samples were obtained in a flow of pure nitrogen ($N_{2}$). Analysis of these dependencies confirmed the fundamental influence of porosity on the electrical behavior of the structure. Weak frequency dispersion was observed for the monocrystal (MC): the impedance modulus $\left|Z_{0}\right|$ at a frequency of 1 kHz was 152.3 ± 2.1 kΩ and decreased to 145.8 ± 1.9 kΩ at 100 kHz, which corresponds to almost resistive behavior with a slight parasitic capacitance. The phase angle $\theta_{0}$ fluctuated between −5° to −8° (Table 2).

### 2.3. Porous GaAs Fabrication

In the conducted experiments, the main controlled morphological parameter was volumetric porosity P, defined as the ratio of pore volume to the total layer volume. Porous GaAs layers were fabricated by anodic electrochemical etching in an ${HF:C}_{2}H_{5}OH$ electrolyte with a volume ratio of 1:3. The principal technological parameter used to control porosity was the anodization current density, which was varied systematically from 10 to 75 mA cm^−2^. Etching was performed at a constant illumination voltage and an electrolyte temperature of 22 ± 1 °C. Under these conditions, the current density determined the final degree of porosity and enabled the preparation of a graded sample set suitable for morphology–response analysis. The reference monocrystal was intentionally left unetched to isolate the influence of the porous matrix itself.

The SEM analysis confirms the evolution of the porous morphology across the investigated sample series. At the same time, the present work focuses on porosity-dependent electrical response rather than on a complete reconstruction of pore topology. Therefore, porosity P, together with the measured structural descriptors in Table 1, is used as the primary morphology parameter for correlating material structure with impedance response.

### 2.4. Sensor Structure and Contact Formation

Ohmic contacts were formed using an AuGe/Ni/Au metallization stack (200/50/150 nm) deposited by thermal evaporation followed by annealing. The contact geometry ensured stable electrical coupling and uniform current distribution across the sensing area. The contact resistance remained low and reproducible across the sample series, increasing gradually from 1.5 ± 0.2 Ω for P10 to 6.0 ± 0.7 Ω for P70. This trend is consistent with increasing structural discontinuity and reduced effective conductivity at higher porosity.

### 2.5. Experimental Setup and Automated Measurement System Details

The measurement platform comprised a hermetic gas chamber, a programmable gas-mixing and delivery unit, an impedance analyzer based on the AD5933 integrated circuit, and a supervisory automation layer implemented through a Siemens S7-1200 programmable logic controller and LabVIEW-based control software. The gas chamber was manufactured from stainless steel, had an internal volume of 50 cm^3^, and was equipped with gas inlet/outlet ports, a type-K temperature sensor, and an absolute pressure sensor. The sample holder incorporated a heating element used for temperature stabilization and post-measurement recovery.

Impedance measurements were performed using a modified Eval-AD5933-EBZ evaluation board (Analog Devices, Inc., Norwood, MA, USA) based on the AD5933 impedance converter. The standard calibration resistor was replaced with a connector for the sensor cell, enabling direct measurement of the GaAs samples. The operating range was 1 kHz to 100 kHz, with the useful impedance interval lying between approximately 1 kΩ and 10 MΩ. Although the AD5933 is not a full laboratory impedance workstation, it is increasingly used in compact and automated sensing platforms, provided that calibration and averaging procedures are carefully implemented [6,9]. In the present system, low-level communication, scan control, data acquisition, and logging were performed through LabVIEW virtual instruments, while the PLC handled temperature control and gas-delivery logic. This architecture is scientifically relevant because it enabled synchronized gas-environment control and multi-frequency impedance acquisition rather than merely instrument automation.

### 2.6. Gas Exposure Protocol

The experimental procedure for each sample began with its mounting in a specialized hermetic measuring chamber with stabilized platinum contacts. After mechanical fixation and a tightness check, the automated hardware-software complex was started. The first stage was performing the calibration procedure for the AD5933-based impedance analyzer. After calibration, the system was switched to the baseline state stabilization mode. The chamber was filled with a flow of pure nitrogen (N_2_, 5.0) at a rate of 200 ± 2 mL·min^−1^, which provided about 10 full gas exchanges per minute. The temperature was stabilized at 25.0 ± 0.2 °C using the PLC’s PID controller, which managed the heating element integrated into the sample mounting base. The criterion for reaching a stable baseline state was a change in the impedance modulus $\left|Z_{0}\right|$, measured at a reference frequency of 5 kHz, by less than 0.25% within 15 min. This stage usually lasted 60–90 min and allowed for the desorption of residual gases and the balancing of thermal stresses.

The main stage was the execution of the concentration cycle. The main LabVIEW script set a sequence of target nitrogen dioxide concentrations $C_{target}$: 0 → 1 → 5 → 10 → 25 → 50 → 100 → 50 → 10 → 5 → 1 → 0 ppm. After changing the gas environment, the system entered standby mode for response stabilization. The value of |Z| was software-monitored at a frequency of 5 kHz, and the transition to measurement occurred only under the condition:

$$\frac{\left|Z\left(t\right)-Z(t-∆t)\right|}{Z(t-∆t)}<0.1\%$$

for a period of ∆t=60 s. This ensured that the frequency sweep was performed under near-equilibrium adsorption conditions. After stabilization, a full frequency scan was launched. The AD5933 generator formed a sinusoidal voltage with an amplitude of 500 mV, and for each of the 50 frequencies in a logarithmically uniform range from 1 kHz to 100 kHz, a 64-fold averaging of the DFT results was performed to reduce noise. Associated environmental parameters were simultaneously recorded: temperature T, absolute pressure p in the chamber, relative humidity RH (<5%) and the calculated $C_{actual}$.

The final stage of the protocol for each sample was the recovery procedure. After the completion of the concentration cycle, a prolonged (90 min) purging of the chamber with pure $N_{2}$ at an elevated temperature of 80 °C was performed. This ensured almost complete desorption of ${NO}_{2}$ molecules and the return of the impedance to its original baseline value $Z_{0}$ with an error of no more than 2%, which confirmed the reversibility of the process and the readiness of the system for the next experiment or sample change. To assess repeatability, the full measurement cycle was repeated three times for each sample. The data were then processed and visualized using LabVIEW 2026 community edition and MATLAB v2023b. 

### 2.7. Impedance Measurement Procedure

Once the gas atmosphere reached the target concentration, the system remained in a stabilization state until the impedance at 5 kHz approached steady behavior. The transition to the frequency sweep was performed only after a software stability criterion had been satisfied for a defined holding period. Each measurement cycle was repeated three times for all samples, and the results are reported as mean values with corresponding deviations. The statistical analysis is therefore intended to demonstrate repeatability and consistency of the observed trends rather than to provide high-power inferential statistics. This approach is sufficient for evaluating the influence of porosity on impedance response under controlled experimental conditions.

The frequency scan consisted of 50 logarithmically distributed points between 1 kHz and 100 kHz. A sinusoidal excitation of 500 mV was applied, and each frequency point was obtained from 64-fold averaging of the DFT output to suppress noise. For each scan, the real and imaginary components of impedance, the impedance modulus |Z|, and phase angle φ were recorded together with gas concentration, temperature, pressure, and humidity. Based on both the theoretical pre-analysis and the experimental results, particular emphasis was placed on the low-frequency region around 5–10 kHz, where the response to NO_2_ was strongest.

### 2.8. Data Processing and Parameter Extraction

Primary data processing included conversion of the AD5933 output into calibrated impedance spectra, low-pass filtering with a Hamming-window-based routine, and averaging over repeated acquisition cycles. The principal observables used for analysis were |Z|, φ, the real and imaginary impedance components, and Nyquist-plot features. A fixed reference frequency of 5 kHz was selected for concentration–response analysis on the basis of the experimentally derived frequency-dependent sensitivity curves *S*(*f*) presented in Section 3.2. These curves demonstrate that 5 kHz falls within the sensitivity maximum region for all investigated samples, providing the highest relative impedance response while remaining sufficiently above the low-frequency noise floor of the AD5933-based analyzer. The selection is therefore grounded in both experimental evidence and measurement system constraints.

To quantify the sensor response, the dependence of the impedance modulus |Z|, measured at a fixed frequency of 5 kHz, on the ${NO}_{2}$ concentration $C_{gas}$ was obtained for each sample (Table 3).

All dependencies had a pronounced nonlinear, sigmoidal character, well described by the modified Langmuir-Freundlich equation integrated with the exponential barrier model:

(1) |Z|(C)=|Z0|+∆|Z|max·((K·C)ν(1+(K·C)ν)).

The relative sensitivity $S_{rel}$, defined as:

(2) $$S_{rel}=\;\frac{\left|Z(C=10\;ppm)\right|-\;\left|Z_{0}\right|}{\left|Z_{0}\right|}\;\cdot 100\%,$$

demonstrated a non-monotonic dependence on the degree of porosity P.

As seen from Table 4, the sensitivity increased from 46.2% for P10 to a maximum value of 205.3% for the sample P50, after which a slight decrease to 187.5% was observed for P70, which is fully consistent with theoretical predictions regarding the existence of an optimal porosity value. Response time, $\tau_{90}$ represents the time to reach 90% of the full response under a concentration step from 0 to 10 ppm. Recovery time, $\tau_{rec}$ represents the time to return to 110% of the initial value $\left|Z_{0}\right|$ after purging. The hysteresis area $A_{hyst}$ was extracted from the difference between the ascending and descending concentration branches.

### 2.9. Equivalent-Circuit Fitting and Parameter Extraction

To quantitatively validate the proposed physical–electrical model and to extract numerical parameters describing the sensor response, a full approximation of the experimental impedance spectra was performed using nonlinear least-squares fitting.

The fitting procedure was implemented in MATLAB R2023b using the *lsqnonlin* function based on the Levenberg–Marquardt algorithm. The optimization minimized the combined residuals of the real $Z^{\prime }$ and imaginary $Z^{\prime \prime }$ components of the impedance across all measured frequencies simultaneously.

**Figure 3:**
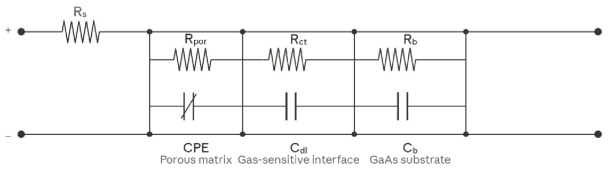
Equivalent-circuit model used for fitting impedance spectra of porous GaAs gas-sensing structures. The model consists of a series resistance *R*_s_, a porous-matrix branch *R*_por_||CPE, a gas-sensing interfacial branch *R*_ct_||C_dll_, and a bulk substrate branch *R*_b_||*C*_b_.

The equivalent circuit adopted in this study consists of a series bulk resistance *R*_s_ representing contact and substrate contributions, followed by a parallel *R*_por_||CPE sub-circuit accounting for the distributed resistive-capacitive response of the porous matrix, and a parallel *R*_ct_||*C*_dl_ sub-circuit representing charge-transfer resistance and double-layer capacitance at the gas-sensitive interface, with an additional parallel *R*_b_||*C*_b_ element describing the contribution of the GaAs substrate bulk. The full topology is therefore: *R*_s_—(*R*_por_||CPE)—(*R*_ct_||*C*_dl_)—(*R*_b_||*C*_b_) (Figure 3).

To validate the choice of this topology, three alternative circuit models were evaluated against the experimental spectra:

(i) A simplified single-relaxation model *R*_s_ + (*R*_ct_||CPE);
(ii) A double-relaxation model *R*_s_ + (*R*_por_||CPE1) + (*R*_ct_||CPE2);
(iii) The selected model augmented with a Warburg diffusion element *W* in series with *R*_ct_.

Model selection was performed using the Akaike Information Criterion (AIC) corrected for finite sample size (AICc), calculated from the weighted residual sum of squares and the number of free parameters. The selected four-element model provided the most physically meaningful description of the experimental spectra values across all samples and concentrations, with ΔAIC > 4 relative to the single-relaxation model and ΔAIC < 2 relative to the double-CPE variant, indicating that the added complexity of the double-CPE model was not statistically justified. The Warburg-augmented model did not yield a statistically significant improvement in fit quality (*p* > 0.05 based on F-test), and the available experimental data did not justify inclusion of an explicit diffusion element for most samples, confirming that explicit diffusion elements were not required by the data within the measured frequency range of 1–100 kHz. The selected circuit is therefore the most parsimonious model consistent with the physical structure of the porous GaAs sensing element.

The objective function F(θ) was defined over all frequency points $f_{k}(k=1\ldots 50)$ as:

$$F\left(\theta \right)=\left[\begin{array}{c} \begin{array}{c} \frac{Z_{exp}^{\prime }(f_{1})-Z_{mod}^{\prime }(f_{1})}{\sigma_{Z^{\prime }}}\\ \frac{Z_{exp}^{\prime \prime }(f_{1})-Z_{mod}^{\prime \prime }(f_{1})}{\sigma_{Z\prime \prime }} \end{array}\\ \vdots \\ \frac{Z_{exp}^{\prime }(f_{50})-Z_{mod}^{\prime }(f_{50})}{\sigma_{Z^{\prime }}}\\ \frac{Z_{exp}^{\prime \prime }(f_{50})-Z_{mod}^{\prime \prime }(f_{50})}{\sigma_{Z\prime \prime }} \end{array}\right]$$

where *θ* = [*R*_s_, *R*_por_, *Q*, *n*, *R*_ct_, *C*_dl_, *R*_b_, *C*_b_] is the vector of equivalent-circuit parameters. The weighting coefficients $\sigma_{Z\prime }$ and $\sigma_{Z\prime \prime }$ were set to 1% of the maximum value of the corresponding impedance component, ensuring balanced contributions from all frequency regions.

Initial parameter estimates were selected based on preliminary analysis (*R_s_* = 5.3 Ω, *R*_por_ = 8.7 kΩ, *Q* = 83.5 × 10^−9^, *n* = 0.8, *R*_ct_ = 28.05 kΩ, *C*_dl_ = 9.7 nF, *R*_b_ = 9.9 kΩ, *C*_b_ = 94 pF). Parameter bounds were imposed to ensure physical consistency (e.g., 0 < *n* < 1, all resistances positive).

For each fitted spectrum, the reduced chi-square (*χ*^2^) was calculated, and confidence intervals for the fitted parameters were estimated from the Jacobian matrix at the solution point.

For the representative sample P50 at *C*_NO2_ = 10 ppm, the fitting procedure yielded a reduced chi-square of 1.85 × 10^−3^, with a maximum relative residual below 2.9% and an average residual of 1.23%, indicating high-quality agreement between model and experiment.

The fitting results for representative samples (MC, P30, P50, and P70) at selected NO_2_ concentrations of 0, 10, and 50 ppm are summarized in Table 5. This reduced but representative dataset was selected to cover the reference monocrystalline sample, an intermediate-porosity sample, the sample with maximum relative response, and the high-porosity limit. The purpose of this analysis was to verify whether the fitted parameters evolve in a physically consistent manner and whether the NO_2_-induced response can be attributed primarily to interfacial charge-transfer processes rather than to a bulk change in the porous GaAs framework.

For the porous samples, the most pronounced concentration-dependent variation was observed for the charge-transfer resistance (*R*_ct_). In sample P50, for example, (*R*_ct_) increased from 688 kΩ at 0 ppm to 2085 kΩ at 10 ppm and 3840 kΩ at 50 ppm. Over the same concentration range, *R*_por_ changed only from 8.5 to 9.1 kΩ, corresponding to a variation of less than 10%. A similar trend was observed for P30 and P70. This behavior indicates that the NO*_2_-induced impedance response is governed mainly by modulation of the gas-sensitive interfacial component represented by R*_ct_, while the resistance of the porous framework remains comparatively stable.

At the same time, the fitted parameters should not be interpreted as fully independent microscopic observables. Equivalent-circuit fitting of heterogeneous porous systems is intrinsically non-unique, and parameter coupling may occur between resistive and non-ideal capacitive elements. For this reason, the present analysis is used to support a physically consistent interpretation of the impedance response rather than to provide a unique microscopic decomposition. The low reduced χ^2^ values and maximum relative residuals below approximately 4% indicate that the selected model reproduces the representative spectra with acceptable accuracy, but a complete parameter-correlation analysis for all samples and all concentrations is beyond the scope of the present revision.

In Table 5 we used the following legend: C_NO2_—NO_2_ concentration; *R*_s_—series/contact resistance; *R*_por_—porous framework resistance; Q—CPE pseudo-capacitance parameter; *n*—CPE exponent; *R*_ct_—charge-transfer resistance; *C*_dl_—double-layer/interfacial capacitance; *R*_b_ and *C*_b_—bulk substrate branch; *χ*^2^_red_—reduced chi-square.

The dash indicates that the corresponding interfacial adsorption-related parameter was not resolved for the monocrystalline reference under the selected fitting topology.

### 2.10. Statistical Treatment and Reproducibility

To evaluate the reproducibility of the experimental results, each measurement cycle was repeated three times for all samples under identical conditions. The reported values are presented as mean ± deviation, calculated from these repeated measurements. This approach enables assessment of measurement stability and consistency across the investigated porosity range.

The statistical treatment in this study is focused on repeatability analysis rather than on inferential statistical testing. Given the controlled experimental conditions and the primary objective of identifying systematic trends in impedance response as a function of porosity and gas concentration, this level of analysis is sufficient to support the observed relationships.

## 3. Results

### 3.1. Baseline Impedance Behavior in Nitrogen

The present study focuses on the NO_2_ response; selectivity toward other gases was not investigated. Baseline measurements in pure nitrogen showed that porosity strongly affected the electrical response even in the absence of NO_2_. The impedance spectra depended on excitation frequency, sample porosity and gas concentration, confirming that the porous layer introduced a morphology-dependent electrical response (Table 2).

This trend indicates that the porous layer introduced an increasingly non-ideal and capacitive response. For the highest-porosity sample, the low-frequency impedance was markedly elevated, reaching 1.12 ± 0.08 MΩ at 1 kHz and decreasing to 124.5 ± 7.5 kΩ at 100 kHz. The phase minimum for porous samples was observed in the approximate range of 10–30 kHz, suggesting the presence of distributed relaxation processes rather than a single ideal RC response. The depth of this phase minimum increased with porosity, consistent with stronger heterogeneity of the porous matrix and a more important role of interfacial polarization.

### 3.2. Frequency-Dependent Response Under ${NO}_{2}$ Exposure

**Figure 4:**
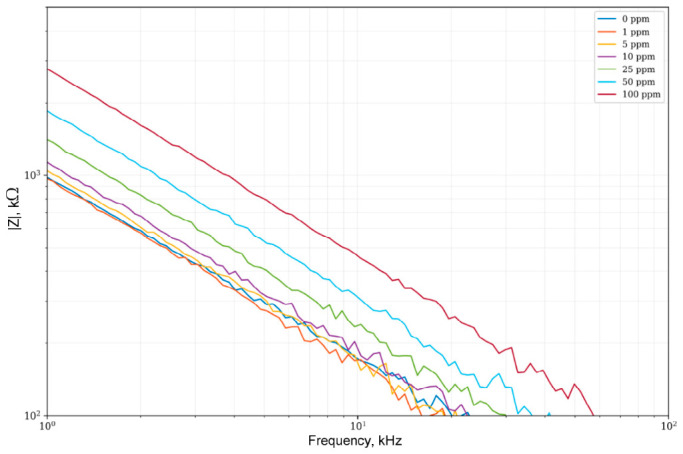
Frequency dependence of the impedance magnitude |Z| for sample P50 under NO_2_ exposure. Spectra were recorded in nitrogen-based gas mixtures at NO_2_ concentrations from 0 to 100 ppm over the frequency range of 1–100 kHz using a 500 mV sinusoidal excitation. Measurements were performed after stabilization of the impedance response at each concentration.

Exposure to NO_2_ shifted the impedance spectra upward over the full measured frequency range, with the strongest effect observed at low frequencies. For the representative P50 sample, the impedance curves (|*Z*|(*f*)) recorded between 0 and 100 ppm NO_2_ preserved the general monotonic decrease of (|*Z*|) with frequency, but the separation between curves was largest at the low-frequency end (Figure 4). At 0 ppm, |Z| changed from 985 ± 22 kΩ at 1 kHz to 185 ± 4 kΩ at 100 kHz, whereas with increase in $C_{gas}$ to 100 ppm the corresponding 1 kHz value increased to 2.85 ± 0.12 MΩ. Above approximately 50 kHz, the concentration-dependent separation became much smaller. This indicates that the ${NO}_{2}$-induced response was governed mainly by low-frequency processes associated with interfacial charge transfer.

The concentration-dependent increase in |Z| was therefore not uniform across the frequency axis. Instead, it was strongly weighted toward the spectral region in which interfacial and adsorption-related processes dominate the impedance response. This observation is one of the central experimental outcomes of the study because it supports the use of impedance spectroscopy, and especially low-frequency analysis, as a more informative approach than single-point resistance measurement alone.

**Figure 5:**
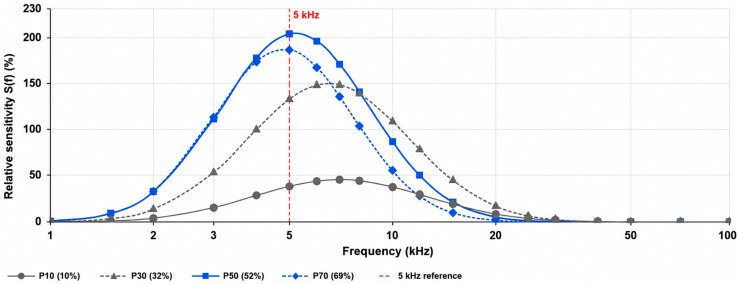
Frequency-dependent relative sensitivity *S*(*f*) = [*Z*(*f*, *C*) − *Z*(*f*, 0)] / *Z*(*f*, 0) × 100% for porous GaAs samples with different porosity levels. The curves were calculated at C_NO_2__ = 10 ppm using the corresponding nitrogen baseline as |*Z*(*f*, 0)|. The maximum sensitivity occurs in the low-frequency region of approximately 5–10 kHz, supporting the selection of 5 kHz as the reference frequency for concentration–response analysis.

To quantitatively characterize the frequency dependence of the sensing response and to justify the selection of the reference measurement frequency, the relative sensitivity *S*(*f*) was calculated across the full measured frequency range for each porous sample according to:

*S*(*f*) = [*Z*(*f*, *C*) − *Z*(*f*, 0)]/*Z*(*f*, 0) × 100%

where *C* = 10 ppm was used as the reference NO_2_ concentration. The resulting *S*(*f*) curves are shown in Figure 5. For all porous samples, the sensitivity exhibited a pronounced maximum in the low-frequency region, with the peak located between 5 and 10 kHz. Above this range, *S*(*f*) decreased monotonically with increasing frequency, reaching values below 15–20% of the peak sensitivity at 100 kHz. The frequency of maximum sensitivity shifted slightly toward lower values with increasing porosity, consistent with a progressively slower dominant relaxation process in more heterogeneous structures. These results establish that 5 kHz lies within the region of highest sensitivity for the entire sample series and provide a physically and statistically grounded basis for its selection as the reference frequency for concentration-dependent analysis.

### 3.3. Concentration Dependence of Impedance

At the reference frequency of 5 kHz, the dependence of |Z| on NO_2_ concentration was nonlinear for all porous samples and followed a sigmoidal trend (Table 3). These data were fitted with a modified Langmuir–Freundlich-type expression (1), and it was found that the apparent sensitivity constant K increased monotonically with porosity, from 0.12 ± 0.02 ppm^−1^ for P10 to 0.38 ± 0.03 ppm^−1^ for P70, confirming an increase in adsorption efficiency. At the same time, the heterogeneity exponent ν decreased slightly from 0.75 ± 0.05 to 0.65 ± 0.05, suggesting increasingly distributed adsorption or electrical environments at higher porosity. The estimated sensitivity threshold, which represents the concentration at which ∆|Z| reaches 10% of ${∆\left|Z\right|}_{max}$, improved from approximately 15 ppm for P10 to approximately 2 ppm for P70 (Table 3).

Although the fitted parameter K increased monotonically, the normalized response did not. The concentration–response curves for intermediate-porosity samples rose more steeply in the practically relevant low-to-moderate concentration range, while the highest-porosity samples combined a larger baseline impedance with stronger saturation and slower recovery. Thus, the concentration dependence of |Z| indicates that increasing porosity enhanced adsorption-related interaction with NO_2_, but did not improve all sensor metrics simultaneously.

### 3.4. Influence of Porosity on Sensitivity and Kinetics

The effect of porosity was particularly clear when the response at 10 ppm NO_2_ was compared across the sample series (Table 4). Relative sensitivity increased from 46.2 ± 2.9% for P10 to 150.3 ± 5.8% for P30 and reached a maximum of 205.3 ± 6.2% for P50. At still higher porosity, the response decreased slightly to 187.5 ± 7.5% for P70. Thus, the experimental optimum occurred near 52% porosity, in close agreement with the model prediction of an optimum near 53.1%.

The kinetic behavior changed concurrently with sensitivity. Response time increased from 12.5 ± 1.5 min for P10 to 18.2 ± 2.1 min for P50 and 22.5 ± 2.5 min for P70, while recovery time increased even more markedly, from 15.2 ± 2.0 min to 35.1 ± 3.8 min and 48.3 ± 5.1 min for the same samples. Therefore, the porosity that maximized sensitivity did not minimize kinetics. Instead, higher porosity tended to increase the interaction strength and total uptake of NO_2_, but at the cost of slower desorption and stronger memory effects.

### 3.5. Nyquist-Plot Features and Equivalent-Circuit Trends

**Figure 6:**
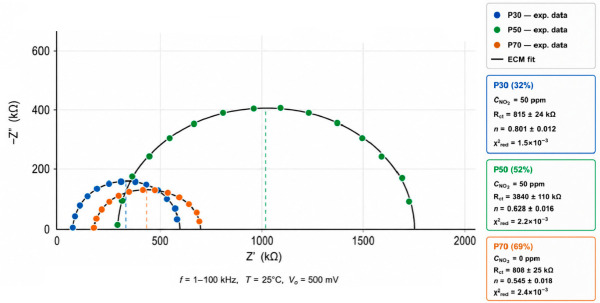
Nyquist plots (−Z″ versus $Z^{\prime }$) for representative porous GaAs samples (P30, P50, P70) under NO_2_ exposure. The spectra were recorded after a stabilization in a nitrogen-based gas mixture containing 100 ppm NO_2_ over the frequency range of 1–100 kHz using a 500 mV sinusoidal excitation. The flattened arcs indicate non-ideal capacitive behavior and distributed relaxation processes in the porous structures.

The Nyquist plots have the form of flattened semicircular arcs rather than ideal semicircles. This departure from ideality became more evident as porosity increased and is consistent with distributed relaxation behavior (Figure 6).

For the P50 sample, the arc diameter ($∆Z^{\prime }\approx 1.45\;MOhm$) was substantially larger than for P30 ($∆Z^{\prime }\approx 0.52\;MOhm$) and slightly smaller than for P70 ($∆Z^{\prime }\approx 1.62\;MOhm$), indicating that the absolute resistive contribution continued to grow with porosity even though the normalized sensitivity had already passed its maximum. The shift of the arc centers along the real-axis direction ($Z^{\prime }$) with the increasing P further indicates that morphology altered the resistive baseline of the porous matrix resistance $R_{por}$ in addition to changing its gas-induced modulation.

In qualitative terms, the Nyquist behavior supports an equivalent-circuit picture involving a resistive porous framework, a gas-sensitive interfacial component, and a non-ideal capacitive element represented by a CPE. The observed flattening of the arcs is consistent with the electrical heterogeneity expected in porous GaAs and provides experimental support for moving beyond a simple ideal R || C description.

The evolution of the CPE parameters extracted from equivalent-circuit fitting provides additional insight into the morphology-dependent electrical response. The CPE exponent n decreased systematically with increasing porosity, from 0.82 ± 0.03 for P30 to 0.55 ± 0.04 for P70 at zero NO_2_ concentration, reflecting the growing electrical heterogeneity of the porous matrix as the pore network becomes more developed and structurally irregular. Upon NO_2_ exposure, n decreased further for all samples, with the most pronounced change observed for P50 and P70, where n approached 0.5 at concentrations above 50 ppm. As discussed in Section 4.3, this is interpreted as a signature of increasingly distributed relaxation times rather than strict diffusion-limited behavior. The pseudo-capacitance parameter *Q* increased monotonically with porosity and showed a strong correlation with the BET-derived specific surface area (*r* = 0.98), consistent with the expected scaling of interfacial pseudo-capacitance with active surface development. Under NO_2_ exposure, *Q* increased further for all porous samples, which is attributed to non-uniform filling of pores of different sizes and the associated broadening of the local capacitive environment distribution. These trends confirm that the CPE parameters are sensitive indicators of both morphological state and gas-induced interfacial changes, and that their systematic evolution across the sample series is physically meaningful rather than an arbitrary outcome of the fitting procedure.

### 3.6. Nonlinear and Hysteretic Response Characteristics

A major result of the automated concentration-cycling protocol was the observation of nonlinear and hysteretic behavior (Table 4). The ascending and descending concentration branches did not overlap fully, and the hysteresis area increased strongly with porosity. At 10 ppm and 5 kHz, the hysteresis area increased from 2.1 ± 0.3 kΩ·ppm for P10 to 6.5 ± 0.8 kΩ·ppm for P30, 14.8 ± 1.2 kΩ·ppm for P50, and 18.7 ± 1.5 kΩ·ppm for P70. Thus, the same structural changes that enhanced low-frequency sensitivity also increased response memory and delayed recovery.

**Figure 7:**
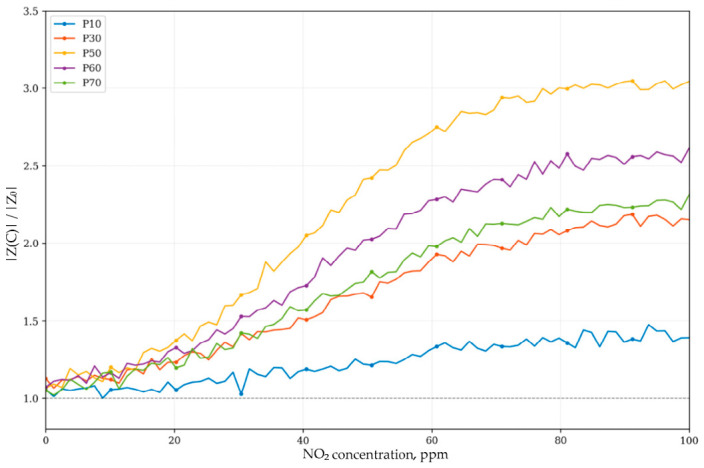
Concentration dependence of the normalized impedance response (|Z|(C)|Z0|) for porous GaAs samples at the reference frequency of 5 kHz. Measurements were performed during the NO_2_ concentration cycle in nitrogen from 0 to 100 ppm. Dashed lines represent the sigmoidal fitting curves used to guide the concentration-response interpretation. The strongest normalized response observed for the intermediate-porosity sample P50.

The concentration–response curves also showed clear saturation at high concentration (Figure 7).

Figure 7 shows nonlinear sigmoidal concentration–response curves for all porous samples. The P50 sample exhibited the largest normalized response: at $C_{{NO}_{2}}=100\;ppm$.

Figure 7 clearly shows a family of sigmoidal curves. The curve for P50 is located highest, demonstrating the greatest relative growth. It is seen that at $C_{gas}=100\;ppm$, |Z|(C)|Z0| reached approximately 3.05, compared with 1.46 for P10. Although P60 and P70 had higher baseline impedance values, their normalized response was lower than that of P50, confirming the presence of an intermediate porosity optimum.

For the porous samples, the normalized impedance increased rapidly in the low-concentration region and approached a plateau above approximately 50 ppm. This behavior is consistent with the statement that the number of electrically active adsorption sites is finite and that surface coverage approaches saturation as concentration increases. The results, therefore, indicate that the sensor response cannot be described adequately by a purely linear concentration law over the full tested range.

Overall, the experimental data provide a physically consistent picture of porous GaAs as an active impedimetric gas-sensing material. The key result is not only the presence of NO_2_ sensitivity, but also the nonlinear relationship between porosity, analyte concentration, and impedance response. This relationship is essential for interpreting the sensing mechanism and for further optimization of porous GaAs-based gas sensors.

## 4. Discussion

### 4.1. Role of Porosity in Shaping the Impedance Response

The main outcome of this study is that porosity plays a dual role in porous-GaAs ${NO}_{2}$ sensing. On one hand, increasing porosity expands the gas-accessible surface, increases the effective number of adsorption-active sites, and amplifies the contribution of surface-controlled electrical processes. On the other hand, excessive porosity reduces the continuity of the semiconductor framework, increases the complexity of current paths, and enhances electrical heterogeneity. The experimental response maximum near P≈50% is therefore physically reasonable and consistent with the broader behavior of porous sensing materials, in which sensitivity often reflects a trade-off between adsorption capacity and electrical transport efficiency [1,6,7,8,9,10].

The structural data reported in Table 1 reinforce this interpretation. As porosity increased, the specific surface area rose substantially, while the effective dielectric constant decreased and contact resistance increased. These trends suggest that the porous layer became simultaneously more adsorption-active and more electrically dispersed. Up to intermediate porosity, the benefit of enhanced surface interaction dominated. Beyond that range, additional porosity likely increased the resistance of the conduction network and reduced the electrical accessibility of a fraction of the active surface. This explains why the fitted adsorption-related constant K increased monotonically, whereas the normalized sensor response passed through a maximum rather than continuing to rise.

The decline in relative sensitivity beyond the optimal porosity arises from a combination of structural and kinetic factors. Primarily, higher porosities progressively disrupt the continuity of the semiconducting GaAs framework. In accordance with percolation theory, exceeding a critical void fraction severely diminishes the number of continuous conductive pathways. This fragmentation drives up the baseline resistance (*R*_por_) and subsequently suppresses the relative contribution of the gas-sensitive interfacial resistance (*R*_ct_) to the overall impedance [10,19]. This structural breakdown is evidenced by the data presented in Table 2, which shows a decrease in the real impedance component (Z’) at 5 kHz for P60 and P70 compared to P50. Because a high, relatively inert *R*_por_ dominates the baseline, even substantial absolute shifts in *R*_ct_ produces a smaller relative variation in total impedance, which directly corresponds to the sensitivity drop observed between P50 and P70 (Table 4).

Furthermore, extensive void fractions create longer, highly tortuous pore channels that escalate the diffusion resistance for NO_2_ molecules [10]. Under the present equilibrium conditions, a significant portion of the internal surface area likely remains kinetically inaccessible within the stabilization window. As a result, the effective population of active adsorption sites is restricted despite the expanded geometric surface area. This diffusion-limited accessibility is corroborated by the monotonic increase in both response and recovery times from P10 to P70 (Table 4).

Additionally, the stronger hysteresis observed at elevated porosities indicates that gas adsorption increasingly occupies energetically deep or sterically hindered sites [18]. Because desorption from these locations is sluggish, they contribute to absolute impedance changes but fail to fully revert to baseline within the measurement timeframe. This behavior artificially elevates the apparent baseline and compresses the dynamic range of the sensor’s relative response.

These interconnected mechanisms, percolation network fragmentation, restricted inner-surface diffusion, and deep-site hysteresis, clarify why absolute impedance variation scales continuously with porosity, whereas relative sensitivity reaches its maximum near *P* ≈ 50%.

### 4.2. Interpretation of Low-Frequency Sensitivity

The strong concentration dependence observed at low frequencies is consistent with the general understanding of impedimetric gas sensing [6,7,9]. In porous and heterogeneous materials, high-frequency impedance tends to emphasize fast bulk and geometric contributions, whereas low-frequency impedance is more sensitive to interfacial charge transfer, barrier modulation, and slow polarization phenomena. In the present study, the largest curve separation under NO_2_ exposure occurred below approximately 10 kHz, and both the numerical pre-analysis and the experimental results identified 5–10 kHz as the most informative region.

It should be noted that the apparent sensitivity decrease at higher frequencies is accompanied by increasing measurement noise, as visible in Figure 3. This reflects two concurrent effects. The AD5933 performs DFT-based signal acquisition, and at higher frequencies the number of captured signal periods within the fixed acquisition window decreases, reducing effective averaging and increasing variance. Also, parasitic capacitances of the connection leads introduce frequency-dependent phase errors that become significant above 30–50 kHz. Consequently, curves for neighboring NO_2_ concentrations partially overlap at high frequencies not solely because the true impedance values converge, but also because measurement uncertainty becomes comparable to the concentration-induced separation. The *S*(*f*) curves presented in Figure 4 use mean values averaged over three repeated acquisitions to partially suppress this effect. This further reinforces the practical advantage of operating at 5–10 kHz, where both the physical sensitivity and the instrument signal-to-noise ratio are simultaneously favorable.

This behavior can be interpreted as follows. NO_2_, being a strong electron acceptor, modifies the near-surface charge state of GaAs and thereby changes the resistance associated with charge transfer across the porous interface. Such changes are most visible at low frequency, where the measurement has sufficient time to sample slower adsorption-controlled interfacial processes. Similar low-frequency amplification has been reported in other impedimetric gas sensors and is one of the reasons impedance spectroscopy is especially informative for chemically heterogeneous systems [6,18,19].

### 4.3. Equivalent-Circuit Perspective and CPE-Based Non-Ideality

The flattened Nyquist arcs and increasing phase dispersion with porosity strongly support the use of a non-ideal equivalent circuit containing a CPE rather than an ideal capacitor [8,16,17]. In porous GaAs, the physical origin of this non-ideality is plausibly linked to a distribution of local pore geometries, surface states, local electric fields, and conduction pathways through the porous network. Under NO_2_ exposure, this distribution is expected to broaden further because adsorption does not occur identically at all sites. The present data are therefore consistent with a circuit containing at least three functional elements: a resistive contribution from the porous framework, a gas-sensitive interfacial resistance associated with charge transfer or barrier modulation, and a non-ideal capacitive branch that accounts for distributed interfacial polarization.

It must be emphasized, however, that CPE parameters cannot be straightforwardly mapped onto individual morphological descriptors such as surface roughness, pore size, or porosity in a one-to-one manner. The CPE admittance *Y* = *Q*(*jω*)*^n^* integrates contributions from a distributed population of local interfacial environments, so any single structural metric captures only a partial projection of the underlying physical reality. With this caveat in mind, the observed correlations between CPE parameters and the structural data reported in Table 1 are physically informative rather than strictly quantitative assignments.

The pseudo-capacitance parameter *Q* showed a strong monotonic correlation with the BET-derived specific surface area SBET across the sample series (Pearson *r* = 0.98, *p* < 0.001), which is consistent with *Q* reflecting the density of electrochemically accessible interfacial sites rather than a true geometric capacitance. Importantly, *Q* should not be equated with a physical capacitance in the usual sense: its units (*F*·*s*^(*n*−1)^) depend on the exponent *n*, and converting *Q* to an equivalent capacitance requires explicit knowledge of the characteristic frequency of the process, typically taken at the arc maximum of the Nyquist plot.

The CPE exponent *n*, in turn, decreased systematically from approximately 0.82 for P30 to 0.55 for P70, tracking the increase in structural heterogeneity as evidenced by the widening pore size distribution and decreasing effective dielectric constant *ε*_eff_ (Table 1). This trend is qualitatively consistent with the interpretation that a broader distribution of local pore geometries and surface environments produces a broader distribution of relaxation times, manifesting as a lower *n*.

Nevertheless, establishing a fully quantitative link between *n* and specific morphological metrics such as pore size distribution width would require complementary characterization. For instance, by fitting the pore size distribution obtained from BET/BJH analysis to a log-normal model and comparing its width to the distribution of relaxation times implied by the CPE, which is beyond the scope of the present study but constitutes a natural direction for future work.

At the same time, equivalent-circuit fitting should be interpreted cautiously. As discussed in Section 1, CPE-based models are physically plausible but not unique [6,7,8,9,16,17]. The present results support the inclusion of a CPE because the spectra deviate systematically from ideal semicircular behavior and because the deviation increases with porosity.

The proposed equivalent-circuit interpretation, incorporating a constant phase element, provides a physically consistent description of the observed impedance behavior and its dependence on porosity. The equivalent circuit parameters are supported by quantitative fitting results presented in Section 2.10. The non-ideal capacitive response and flattened Nyquist arcs are indicative of distributed interfacial processes and structural heterogeneity within the porous layer, in agreement with established impedance models for heterogeneous systems [6,7,8,9,16,17]. At the same time, it should be noted that equivalent-circuit representations are not unique, and different circuit configurations may yield comparable fits to the experimental data. Therefore, the model is best regarded as a consistent and physically plausible framework for interpreting the impedance response, rather than a definitive identification of a unique underlying mechanism.

A specific interpretive question arises from the observation that the CPE exponent *n* approaches 0.5 at high porosity and high NO_2_ concentration. In the classical impedance literature, *n* = 0.5 is the signature of a semi-infinite Warburg diffusion element, and its appearance is sometimes taken as direct evidence of diffusion-controlled transport. However, this equivalence holds strictly only when the *n* ≈ 0.5 behavior persists across a wide, well-defined frequency range and when the phase angle approaches −45° uniformly. These are conditions that are not fulfilled in the present spectra, where the CPE describes a broadband non-ideal response rather than a narrowband diffusion arc.

The decrease in *n* toward 0.5 observed here is therefore interpreted more conservatively as an indicator of increasingly distributed relaxation times, arising from the growing heterogeneity of the porous network at high porosity and from non-uniform pore filling under strong NO_2_ exposure. This interpretation is supported by the model comparison presented in Section 2.9, where the addition of an explicit Warburg element did not yield a statistically significant improvement in fit quality and the available experimental data did not justify inclusion of an explicit diffusion element. It should be noted, however, that the absence of sub-kilohertz measurements limits the ability to conclusively distinguish between CPE-type heterogeneity and true diffusion-limited behavior, since Warburg contributions would be most visible at frequencies below the measured range. This distinction should be revisited in future work employing broadband impedance spectroscopy.

The representative fitting results in Table 5 support the assignment of the dominant NO*_2_-dependent impedance change to the interfacial charge-transfer branch of the equivalent circuit. In all porous samples included in the fitting analysis, R*_ct_ increased markedly with NO*_2_ concentration, whereas R*_por_, *R*_b_, and *C*_b_ varied only weakly. This trend is consistent with adsorption-induced modulation of the surface barrier and interfacial charge-transfer pathway. However, this conclusion should be interpreted within the limitations of equivalent-circuit analysis. The fitted parameters are model-dependent, and correlations between *R*_ct_, CPE parameters, and other circuit elements cannot be fully excluded. Therefore, the increase in *R*_ct_ is best regarded as a robust model-supported indicator of interfacial response rather than as a uniquely isolated microscopic resistance. A complete global parameter-correlation analysis for every sample and concentration would require a larger supplementary dataset and broadband impedance measurements, which are beyond the scope of the present revision. The present interpretation is therefore intentionally conservative: the equivalent-circuit model provides a physically consistent description of the observed spectra and supports the dominant role of interfacial processes, but it does not exclude secondary contributions from porous-network resistance, diffusion-related dispersion, or parameter coupling.

### 4.4. Origin of Nonlinearity and Hysteresis

The nonlinear concentration dependence and the growth of hysteresis with porosity indicate that the sensing response is governed by adsorption-controlled surface processes rather than by a simple ohmic perturbation. The sigmoidal concentration curves are consistent with finite-site adsorption and progressive surface filling, while the increasing hysteresis area suggests that adsorption and desorption do not follow identical energetic pathways. In porous systems, this can arise from a combination of strong binding at specific sites, delayed redistribution of adsorbates within the pore network, and slow relaxation of surface or interface charge states [18,19]. The larger hysteresis and longer recovery times observed for P50 and P70 are fully consistent with this picture.

The data do not allow a unique decomposition of hysteresis into purely chemical versus purely electrical components. Nevertheless, the observed trends are consistent with an increasing population of energetically nonequivalent adsorption environments as porosity rises. This interpretation also aligns with the decrease in the fitted heterogeneity exponent ν and the increasingly non-ideal spectral shape. Therefore, hysteresis in the present system should not be viewed only as a drawback; it also provides mechanistic information on the distributed adsorption–desorption landscape of porous GaAs.

### 4.5. Comparison with Other ${NO}_{2}$ Sensor Platforms

Compared with contemporary metal-oxide, graphene-based, MOF-derived, and mixed-conductor porous NO_2_ sensors, porous GaAs does not yet represent the state of the art in absolute detection limit or room-temperature ultrafast kinetics [1,2,3,4,5,18,19]. Many oxide and hybrid platforms now operate in the ppb range and often deliver much faster response and recovery, particularly when assisted by heterojunction design, photoactivation, or highly optimized nanostructures [1,2,3,4,5]. In that sense, porous GaAs should not be positioned as an immediate performance leader.

Its value lies elsewhere. First, porous GaAs offers a semiconductor platform in which porosity, interface effects, and charge-transfer behavior can be studied through impedance spectroscopy with comparatively direct electrical interpretation. Second, the observed optimum porosity and strong low-frequency sensitivity provide a mechanistically rich response that is particularly suitable for structure–property studies. Third, the present results connect porous-GaAs sensing with the wider literature on Schottky-junction and impedance-based gas sensors [6,11,12,18,19]. Thus, porous GaAs may be better positioned as an underexplored and physically informative sensing material than as a direct competitor to the very best hybrid chemiresistors.

### 4.6. Practical Implications and Limitations of the Present Study

From a practical viewpoint, the results indicate that porous GaAs with intermediate porosity, around 50%, provides the best compromise between sensitivity and electrical transport. The low-frequency range around 5–10 kHz is particularly suitable for analytical operation, because it concentrates most of the NO_2_-induced impedance variation. The automated measurement system also proved useful for capturing dynamic and hysteretic effects that would likely be missed under simpler equilibrium-only measurement protocols. These features are relevant for future compact impedimetric gas sensors and for multiparametric sensing strategies based on frequency-domain signatures.

A second limitation concerns the frequency range of the impedance measurements. The AD5933-based analyzer employed in this study operates reliably between 1 kHz and 100 kHz, and this range was therefore adopted throughout. The sub-kilohertz region (1 Hz–1 kHz) was not accessible with the present instrumentation without hardware modification: the AD5933 output clock constrains the minimum stable excitation frequency to approximately 1 kHz under the averaging and calibration scheme used here, and operation below this limit produces unacceptable distortion of the DFT output due to insufficient cycles per acquisition window. This is a recognized limitation of compact DFT-based impedance analyzers compared to full laboratory instruments such as potentiostat-based frequency response analyzers.

From a physical standpoint, this restriction is non-trivial. Gas adsorption kinetics, interfacial state filling, mass diffusion within pore channels, and slow polarization relaxation are processes that generally manifest at frequencies below 1 kHz, and their signatures, such as a low-frequency Warburg tail or a second depressed semicircle in the Nyquist plot, would not be visible in the present data. It is therefore possible that the current frequency window captures only the faster interfacial charge-transfer processes, while slower diffusion-limited or trapping-related contributions remain unresolved. The observed approach of the CPE exponent *n* toward 0.5 at high porosity and high NO_2_ concentration (Section 2.9) is suggestive of diffusion-like behavior, but this interpretation cannot be fully confirmed without sub-kilohertz data. Extension of the measurement range to 1 Hz–100 kHz using a laboratory-grade impedance analyzer represents a priority for future work and would allow a more complete separation of the contributing processes.

Several limitations, however, must be stated explicitly. First, selectivity was not investigated comprehensively; the present work focuses on NO_2_ response rather than cross-sensitivity to other oxidizing or reducing gases. Second, humidity was kept low and monitored, but its influence was not systematically studied. Third, long-term drift and aging were not established over extended timescales. Fourth, although SEM cross-sections and BET/BJH-derived parameters confirm the main morphological evolution of the porous layers, the present study does not attempt a full three-dimensional reconstruction of pore topology. Therefore, the mechanistic interpretation is based on porosity, specific surface area, average pore diameter, and impedance-derived parameters rather than on a complete pore-network model. Finally, the equivalent-circuit interpretation is physically plausible and well supported qualitatively, but not unique. These limitations do not invalidate the present conclusions, but they define the scope within which the findings should be interpreted.

A further point concerns the AC excitation amplitude of 500 mV applied during impedance measurements. While such an amplitude might exceed the small-signal linearity condition in ideal Schottky or p–n junctions and potentially perturb the interfacial carrier distribution or barrier height, the architecture of the present system largely mitigates these concerns. Rather than acting as sharp rectifying junctions, the porous GaAs sensors acts as distributed resistive–capacitive networks. Consequently, the applied voltage partitions across multiple serial impedance elements, ensuring that the local voltage drop at any single interface remains substantially below the total excitation amplitude. Furthermore, the high overall impedance of the porous samples, ranging from roughly 185 kΩ (P10) to 803 kΩ (P70) at the 5 kHz reference frequency, restricts the resulting current to the microampere regime, which is characteristic of low-perturbation operation. From a practical standpoint, the AD5933-based analyzer utilized in this study relies on a fixed output amplitude, precluding amplitude-dependent verification within the scope of the current instrumentation. While these structural and electrical factors support the validity of our measurements, formally confirming linearity would require comparative impedance spectra acquired across a range of excitation amplitudes (e.g., 10 to 500 mV) using a laboratory-grade workstation. We recognize this as a necessary step for future investigations, particularly before adapting this methodology to lower-impedance structures or junction-dominated sensing systems.

A significant practical limitation is that all measurements were performed in nitrogen rather than in ambient air. This choice was deliberate: an inert background eliminates the influence of oxygen and humidity, allowing NO_2_-induced effects to be isolated and interpreted unambiguously. Such approach is appropriate for a mechanistic study of this kind. However, it means that the reported sensitivity values cannot be directly transferred to real-world conditions without further validation. In air, oxygen adsorption independently modifies the GaAs surface potential and carrier concentration, while humidity introduces competitive adsorption and additional impedance contributions. Characterization under realistic atmospheric conditions is therefore identified as the primary direction for future work.

## 5. Conclusions

This work examined how the porosity of electrochemically etched GaAs affects its impedance response under NO_2_ exposure. The porous samples showed stronger frequency dispersion than the monocrystalline reference, confirming that the porous layer introduces a distributed electrical response rather than a simple resistive behavior. The most pronounced NO_2_-induced changes occurred in the low-frequency range of 5–10 kHz, which makes this region the most useful for analytical operation.

The sensor response did not increase monotonically with porosity. Although higher porosity increased the available surface area and strengthened adsorption-related effects, excessive porosity also reduced the continuity of the conductive framework. As a result, the best compromise between adsorption capacity and electrical transport was observed at an intermediate porosity of approximately 50%, where the maximum relative sensitivity was obtained.

Equivalent-circuit fitting supports the interpretation that NO_2_ detection is governed mainly by interfacial processes. The strong variation of charge-transfer resistance with concentration, together with the decrease in the CPE exponent at higher porosity, indicates that the response is shaped by surface-barrier modulation and increasing electrical heterogeneity within the porous layer. The nonlinear concentration dependence and growing hysteresis further suggest that adsorption and desorption occur through distributed sites with different relaxation characteristics.

The present study positions porous GaAs as a useful platform for mechanism-oriented impedance gas sensing rather than as a direct competitor to the best-performing hybrid NO_2_ sensors in terms of detection limit or response time. Further work should focus on selectivity, humidity influence, long-term stability, and multi-frequency impedance descriptors for improved analyte discrimination.

## Figures and Tables

**Table 1:** Morphological and electrical parameters of the investigated gallium arsenide samples.

Sample	Degree of Porosity, P (%)	Specific Surface Area, $S_{BET}$ (m^2^/g)	Average Effective Pore Diameter, $d_{por}$ (nm)	Porous Layer Thickness, $d_{layer}$ (nm)	Effective Dielectric Constant, $\varepsilon_{eff}$ (Ellipsometer, 1 kHz)	Contact Resistance, $R_{c}$ (Ohm) (4-Probe)	Additional Characteristics
MC	<1 (monocrystal)	0.05 ± 0.01	– *	–	12.90 ± 0.05	1.2 ± 0.1	Control sample
P10	10.2 ± 1.5	8.7 ± 0.5	18 ± 3	480 ± 20	11.35 ± 0.15	1.5 ± 0.2	Uniform morphology
P20	21.5 ± 2.0	19.3 ± 1.0	20 ± 4	475 ± 15	10.21 ± 0.20	1.8 ± 0.2	–
P30	31.8 ± 1.8	35.1 ± 1.5	22 ± 3	470 ± 20	9.15 ± 0.25	2.2 ± 0.3	Onset of nonlinearities formation
P40	39.5 ± 2.2	52.4 ± 2.0	25 ± 5	465 ± 18	8.34 ± 0.30	2.8 ± 0.3	–
P50	52.3 ± 2.5	78.9 ± 2.5	28 ± 4	460 ± 20	7.02 ± 0.35	3.5 ± 0.4	Optima for percolation
P60	60.7 ± 2.0	105.2 ± 3.0	32 ± 6	450 ± 25	6.11 ± 0.40	4.5 ± 0.5	Pronounced heterogeneity
P70	68.9 ± 3.0	142.5 ± 4.5	35 ± 7	430 ± 30	5.47 ± 0.50	6.0 ± 0.7	Boundary structural stability

* A dash (–) indicates that the corresponding parameter is not applicable.

**Table 2:** Averaged impedance parameters of samples at a reference frequency of 5 kHz in pure $N_{2}$ ($C_{gas}=0\;\mathrm{ppm}$).

Sample	$\left|Z_{0}\right|$ (kΩ)	$Z_{0}^{\prime }$ (kΩ)	$-Z_{0}^{\prime \prime }$ (kΩ)	$\theta_{0}$ (Degrees)	Quality Factor, Q=|−Z″Z′|
MC	148.5 ± 2.0	147.9 ± 2.0	16.2 ± 0.8	−6.3 ± 0.3	0.109
P10	185.3 ± 3.5	172.1 ± 3.2	72.5 ± 2.5	−22.8 ± 0.5	0.421
P20	255.7 ± 5.1	205.4 ± 4.1	152.3 ± 4.8	−36.5 ± 0.6	0.741
P30	385.2 ± 8.8	238.9 ± 5.5	304.1 ± 9.1	−51.9 ± 0.7	1.273
P40	502.6 ± 12.1	260.5 ± 6.3	432.8 ± 12.9	−59.1 ± 0.8	1.661
P50	684.8 ± 15.7	278.1 ± 6.4	624.5 ± 16.2	−66.0 ± 0.8	2.245
P60	725.3 ± 18.2	265.8 ± 6.7	672.9 ± 18.9	−68.5 ± 0.9	2.532
P70	802.4 ± 25.0	251.2 ± 7.8	760.1 ± 24.1	−71.8 ± 0.9	3.026

**Table 3:** Parameters of the concentration dependence $\left|Z\right|(C_{gas})$ at a frequency of 5 kHz.

Sample	$\left|Z_{0}\right|$ (kΩ)	${∆\left|Z\right|}_{max}$ (kΩ)	Constant K (ppm^−1^)	Exponent *ν*	Sensitivity Threshold (ppm)
P10	185.3	85.7 ± 4.1	0.12 ± 0.02	0.75 ± 0.05	15
P30	385.2	310.5 ± 12.8	0.19 ± 0.02	0.72 ± 0.05	8
P50	684.8	1220.4 ± 45.6	0.28 ± 0.03	0.70 ± 0.05	4
P70	802.4	1850.2 ± 92.5	0.38 ± 0.03	0.65 ± 0.05	2

**Table 4:** Sensor characteristics of samples under ${NO}_{2}$ exposure (10 ppm, 5 kHz).

Sample	$\left|Z_{0}\right|$ at 10 ppm (kΩ)	Relative Sensitivity, $S_{rel}$ (%)	Response Time, $\tau_{90}$ (min)	Recovery Time, $\tau_{rec}$ (min)	Hysteresis Area, $A_{hyst}$ (kΩ·ppm)
P10	270.8 ± 5.4	46.2 ± 2.9	12.5 ± 1.5	15.2 ± 2.0	2.1 ± 0.3
P30	578.9 ± 13.4	150.3 ± 5.8	15.8 ± 1.8	22.5 ± 2.5	6.5 ± 0.8
P50	2090.5 ± 62.7	205.3 ± 6.2	18.2 ± 2.1	35.1 ± 3.8	14.8 ± 1.2
P70	2305.8 ± 92.2	187.5 ± 7.5	22.5 ± 2.5	48.3 ± 5.1	18.7 ± 1.5

**Table 5:** Representative equivalent-circuit fitting parameters for selected samples and NO_2_ concentrations.

Sample	*C*_NO2_ (ppm)	*R*_s_ (Ω)	*R*_por_ (kΩ)	*Q* (n*F*·*s*^n−1^)	*n*	*R*_ct_ (kΩ)	*C*_dl_ (nF)	*R*_b_ (kΩ)	*C*_b_ (pF)	*χ* ^2^_red_	Max. Residual (%)
MC	0	4.2 ± 0.2	1.1 ± 0.1	1.15 ± 0.08	0.945 ± 0.008	— *	—	9.8 ± 0.4	102 ± 5	0.9 × 10^−3^	1.2
P10	0	4.8 ± 0.3	12.3 ± 0.7	32.5 ± 1.4	0.822 ± 0.010	384 ± 12	8.6 ± 0.4	9.9 ± 0.5	98 ± 6	1.2 × 10^−3^	2.1
P20	10	4.9 ± 0.3	12.7 ± 0.8	33.8 ± 1.6	0.814 ± 0.011	575 ± 16	8.0 ± 0.5	10.0 ± 0.5	99 ± 6	1.4 × 10^−3^	2.3
P30	50	5.0 ± 0.3	13.0 ± 0.9	35.5 ± 2.0	0.801 ± 0.012	815 ± 24	7.3 ± 0.5	10.1 ± 0.6	97 ± 7	1.5 × 10^−3^	2.5
P40	0	5.2 ± 0.4	8.5 ± 0.6	79.2 ± 3.2	0.673 ± 0.014	688 ± 20	11.4 ± 0.7	9.8 ± 0.5	95 ± 7	1.7 × 10^−3^	2.8
P50	10	5.3 ± 0.4	8.7 ± 0.7	83.5 ± 4.0	0.652 ± 0.015	2085 ± 58	9.7 ± 0.7	9.9 ± 0.6	94 ± 8	2.0 × 10^−3^	2.9
P60	50	5.4 ± 0.4	9.1 ± 0.8	89.8 ± 4.8	0.628 ± 0.016	3840 ± 110	8.2 ± 0.8	10.0 ± 0.6	93 ± 8	2.2 × 10^−3^	3.1
P70	0	6.0 ± 0.5	5.1 ± 0.5	143.8 ± 7.5	0.545 ± 0.018	808 ± 25	14.7 ± 1.0	9.8 ± 0.6	91 ± 9	2.4 × 10^−3^	3.3

* A dash (—) indicates that the corresponding interfacial adsorption-related parameter was not resolved for the monocrystalline reference under the selected fitting topology.

## Data Availability

The data supporting the findings of this study are available from the corresponding author upon reasonable request.
